# Electromagnetic situation awareness and modeling for space–air–ground integrated networks

**DOI:** 10.1093/nsr/nwaf492

**Published:** 2025-11-10

**Authors:** Jianping An, Yi Tao, Xiaorui Zhang, Zizheng Hua, Shuai Wang, Gaofeng Pan, Xuanhe Yang, Dusit Niyato, George K Karagiannidis

**Affiliations:** School of Cyberspace Science and Technology, Beijing Institute of Technology, Beijing 100081, China; School of Cyberspace Science and Technology, Beijing Institute of Technology, Beijing 100081, China; School of Cyberspace Science and Technology, Beijing Institute of Technology, Beijing 100081, China; School of Cyberspace Science and Technology, Beijing Institute of Technology, Beijing 100081, China; School of Cyberspace Science and Technology, Beijing Institute of Technology, Beijing 100081, China; School of Cyberspace Science and Technology, Beijing Institute of Technology, Beijing 100081, China; School of Cyberspace Science and Technology, Beijing Institute of Technology, Beijing 100081, China; College of Computing and Data Science, Nanyang Technological University, Singapore 639798, Singapore; Department of Electrical & Computer Engineering, Aristotle University of Thessaloniki, Thessaloniki 54124, Greece

**Keywords:** agent-based modeling, artificial intelligence, complex electromagnetic environment modeling, situation awareness, space–air–ground integrated network, spectrum sensing

## Abstract

The ever-thriving advancement of the space–air–ground integrated network (SAGIN) has driven global connectivity toward greater heterogeneity, spurring a growing demand for spectrum resources. This evolution fosters the formation of the complex electromagnetic environment (CEME), leading to unprecedented challenges in managing and modeling the dynamic and diverse electromagnetic landscape. In response, this survey aims to provide the first comprehensive review of electromagnetic situation awareness and modeling in fields such as cognitive radio, integrated sensing and communication. Specifically, we summarize the evolution of spectrum situation awareness and the application of artificial intelligence in this field. Based on the generated spectrum situation map, we propose a comprehensive modeling method for dynamic and in-depth analysis of electromagnetic environments. Furthermore, we analyze and establish CEME models for diverse scenarios in SAGINs, considering factors like attenuation and interference. Finally, our exploration leads to the identification of promising future research directions, including technology development, specific communication scenarios and actual demand for covert communication, shedding some light on new insights and perspectives for advancing electromagnetic environment sensing and modeling.

## Teaser

This paper presents a comprehensive framework for modeling complex electromagnetic environments, paving the way for optimized and reliable communication in future 6G space–air–ground integrated networks.

## INTRODUCTION

With the rapid development of the second- to sixth-generation (6G) wireless communication technology [1], the global communication system has gradually formed a highly heterogeneous and interconnected framework, such as the space–air–ground integrated network (SAGIN) [2], and the demand for spectrum resources has continued to grow. This directly promotes the formation and evolution of the complex electromagnetic environment (CEME) [3]. The CEME-aware SAGIN aims to optimize the performance of wireless communication systems by incorporating environmental awareness, particularly in multi-layered and multi-dimensional network settings. This approach not only enhances system resource utilization, but also mitigates the adverse effects of electromagnetic interference on communication quality. The following three interrelated factors mainly drive the formation of CEME in vigorously developing SAGINs.

1. *Rapid increase and diversification of communication nodes.* The structural complexity of communication systems has expanded significantly from traditional terrestrial platforms to include space and aerial platforms such as satellites and unmanned aerial vehicles (UAVs). This expansion has led to a rapid increase in the number and variety of communication nodes, creating complex three-dimensional communication scenarios within the SAGIN framework [4].
2. *Increasingly complex electromagnetic propagation environments.* Phenomena such as reflection, diffraction and interference across different spatial domains exacerbate the complexity of signal propagation, and the dynamic distribution of frequency bands in space and time domains further heightens uncertainty.
3. *Diversified and composite functions of electromagnetic signals.* The function of electromagnetic signals evolves from single-purpose use (e.g. communication, remote sensing or navigation) to multifunctional convergence and integrated applications and thus intensifies the challenges of signal management and demands higher accuracy in electromagnetic environment modeling.

These intertwined factors highlight the unprecedented challenges in managing and modeling CEME, emphasizing the need for innovative solutions to address the dynamic and diverse demands of future communication systems.

However, as electromagnetic environments grow increasingly complex, traditional modeling methods struggle to accurately capture dynamic, multi-dimensional interactions. To address this challenge, accurate information extraction from the electromagnetic environment is achieved through situational awareness and prediction. This enables a deeper understanding of the actual state of CEME. Building on this foundation, dynamic modeling of key parameters within the environment is performed, and the accurate construction of the CEME model is facilitated through advanced methods [5,6] such as artificial intelligence (AI), multi-agent learning and digital twin technologies. These approaches collectively enable comprehensive electromagnetic space modeling, providing robust support for the future global wireless communication system.

Our paper focuses on building accurate models of CEME to enable comprehensive electromagnetic spatial perception. Spectrum situation awareness (SA), comprising spectrum sensing, situation completion and prediction, is foundational to this effort [7]. By combining methods such as generative AI (GAI), semantic analysis and signal processing, this approach enables precise information extraction from the electromagnetic environment. Continuous monitoring and prediction of the electromagnetic landscape provide a deeper understanding of its real-time dynamics and evolving patterns, while also supplying crucial spectrum data for subsequent electromagnetic modeling.

By integrating digital twins, agent-based modeling (ABM) and situation modeling methods, a multi-dimensional, multi-level electromagnetic modeling framework emerges that not only captures individual node behaviors, but also optimizes network allocation, significantly enhancing communication performance in CEME. Building on spectrum situation awareness data and integrated modeling approaches, this paper further proposes a scenario-driven analysis incorporating both integrated and distributed SAGIN architectures to examine CEME characteristics across space-, air- and ground-based networks [8]. The approach emphasizes noise sources, interference distribution and signal attenuation across different network tiers. By offering a new perspective on multi-layer network environments, our work lays the foundation for addressing system-level challenges in CEME-aware SAGIN implementation, while advancing CEME modeling and dynamic adaptation mechanisms through quantifiable performance metrics and real-time data fusion strategies, paving the way for future spectrum situational awareness and environmental optimization in cross-space systems.

Future electromagnetic environment modeling faces challenges from more stereoscopic communication scenarios and higher-frequency bands. With the development of SAGIN, it must meet the ultra-low latency and high-reliability requirements for 6G, as outlined by the International Telecommunication Union [9]. Applications like autonomous vehicles, industrial automation and telemedicine require near-instantaneous data transmission and high-quality-of-service standards. Additionally, the expansion of the Internet of Things highlights the need for efficient spectrum utilization across frequency bands, such as millimeter waves and terahertz, to ensure reliable communication-system performance [2].

However, existing CEME modeling methods often lack the accuracy and adaptability needed to capture dynamic changes in complex scenarios. In response to this situation, the next part of this section will address the shortcomings in existing literature and propose a more comprehensive approach to support the successful implementation of 6G and future communication systems.

### Existing literature

Although existing literatures lay a foundation for CEME modeling, critical gaps persist across three main dimensions: spectrum situational awareness advanced modeling methodologies, and analysis within SAGIN. Firstly, Spectrum SA is fundamental to modeling methods in CEME; however, existing research [10] has focused on specific aspects rather than providing a comprehensive overview. Axell *et al.* [11] did not encompass discussions of emerging AI techniques. Integrating AI and semantic analysis into spectrum sensing can overcome limitations in dynamic adaptation and interference detection and can uncover context-aware insights that conventional techniques cannot achieve.

Secondly, modeling methods provide a foundational approach for CEME modeling, but the existing literature often focuses on mathematical models and numerical simulations. Some studies integrate traditional modeling with ABM [12], yet their computational demands and limited ability to capture nonlinear dynamics remain key constraints. More recently, electromagnetic information theory has emerged as a fundamental bridge between electromagnetic modeling and information-theoretic performance evaluation, offering a unified framework to assess communication and sensing capabilities in complex environments [13–15]. However, current research still lacks a comprehensive incorporation of situation modeling and digital twins, which are crucial for real-time simulation and dynamic monitoring of electromagnetic environments [16]. Such an integrated method enables accurate real-time decision-making, dynamic adaptation to complex electromagnetic scenarios and predictive analysis that traditional methods cannot achieve.

Thirdly, the existing literatures have some shortcomings in the analysis of CEME in SAGINs. Many studies have examined signal attenuation and interference across different scenarios, such as space, air and ground communications. However, most of these analyses focus on individual scenarios in isolation, neglecting the interactions and complexities that arise when considering different environments together. While current research in CEME-aware SAGIN systems integrates quantum computing with intelligent game theory to advance multi-agent reinforcement learning and quantum annealing [17], key challenges persist in quantum noise suppression and hardware scalability. In addition, some studies oversimplify the attenuation model and approximate the attenuation process to a single distribution [18], which fails to fully reflect the complexity and diversity of signal attenuation in the actual environment. A more comprehensive approach can address these issues by capturing the intricate interactions across space, air and ground, and accurately modeling the diverse and dynamic nature of signal attenuation, enabling more reliable and robust communication network design.

Table 1 provides a comprehensive comparison between this survey and 14 existing studies across key dimensions of electromagnetic environment modeling. The analysis reveals that, despite progress in individual aspects, current research still lacks the depth and integration required to address the complexity of SAGIN scenarios. In particular, deficiencies remain in areas such as cross-domain interaction modeling and the adoption of realistic attenuation models. These limitations hinder the applicability of existing methods to the highly dynamic and heterogeneous SAGIN environment. Table 1 thus highlights the need for more holistic and adaptive modeling approaches tailored to the unique demands of SAGIN.

**Table 1.** Comparison with the literature.

	Spectrum sensing			Electromagnetic environment modeling methods				Electromagnetic environment modeling for SAGIN				
									Signal interference			
	Signal	Signal	Spectrum	Traditional	Digital twin	Agent-based	Situation cognitive		Air-	Ground-	Space-	
Ref.	detection	recognition	assignment	modeling	modeling	modeling	modeling	Attenuation	based	based	based	Noise
[12]						$\checkmark$				$\checkmark$		
[16]	$\checkmark$	$\checkmark$	$\checkmark$				$\checkmark$					
[19]	$\checkmark$				$\checkmark$					$\checkmark$		$\checkmark$
[20]				$\checkmark$				$\checkmark$		$\checkmark$		$\checkmark$
[21]	$\checkmark$			$\checkmark$				$\checkmark$	$\checkmark$			
[22]								$\checkmark$	$\checkmark$			
[23]	$\checkmark$							$\checkmark$			$\checkmark$	
[24]	$\checkmark$			$\checkmark$								
[25]	$\checkmark$						$\checkmark$					$\checkmark$
[26]	$\checkmark$		$\checkmark$			$\checkmark$						$\checkmark$
[27]	$\checkmark$			$\checkmark$				$\checkmark$		$\checkmark$		$\checkmark$
[28]	$\checkmark$	$\checkmark$	$\checkmark$				$\checkmark$			$\checkmark$		$\checkmark$
[29]	$\checkmark$	$\checkmark$				$\checkmark$	$\checkmark$					
[30]	$\checkmark$		$\checkmark$				$\checkmark$					$\checkmark$
This paper	** $\checkmark$ **	** $\checkmark$ **	** $\checkmark$ **	** $\checkmark$ **	** $\checkmark$ **	** $\checkmark$ **	** $\checkmark$ **	** $\checkmark$ **	** $\checkmark$ **	** $\checkmark$ **	** $\checkmark$ **	** $\checkmark$ **

### Motivations and paper organization

While some early papers have mentioned electromagnetic situation awareness and modeling, it is now necessary to critically evaluate them in light of recent advancements:

- lack of a comprehensive summary of spectrum situation awareness;
- lack of up-to-date research on electromagnetic environment modeling methods;
- lack of robust and overall electromagnetic environment models for different scenarios in SAGIN.

**Figure 1.**
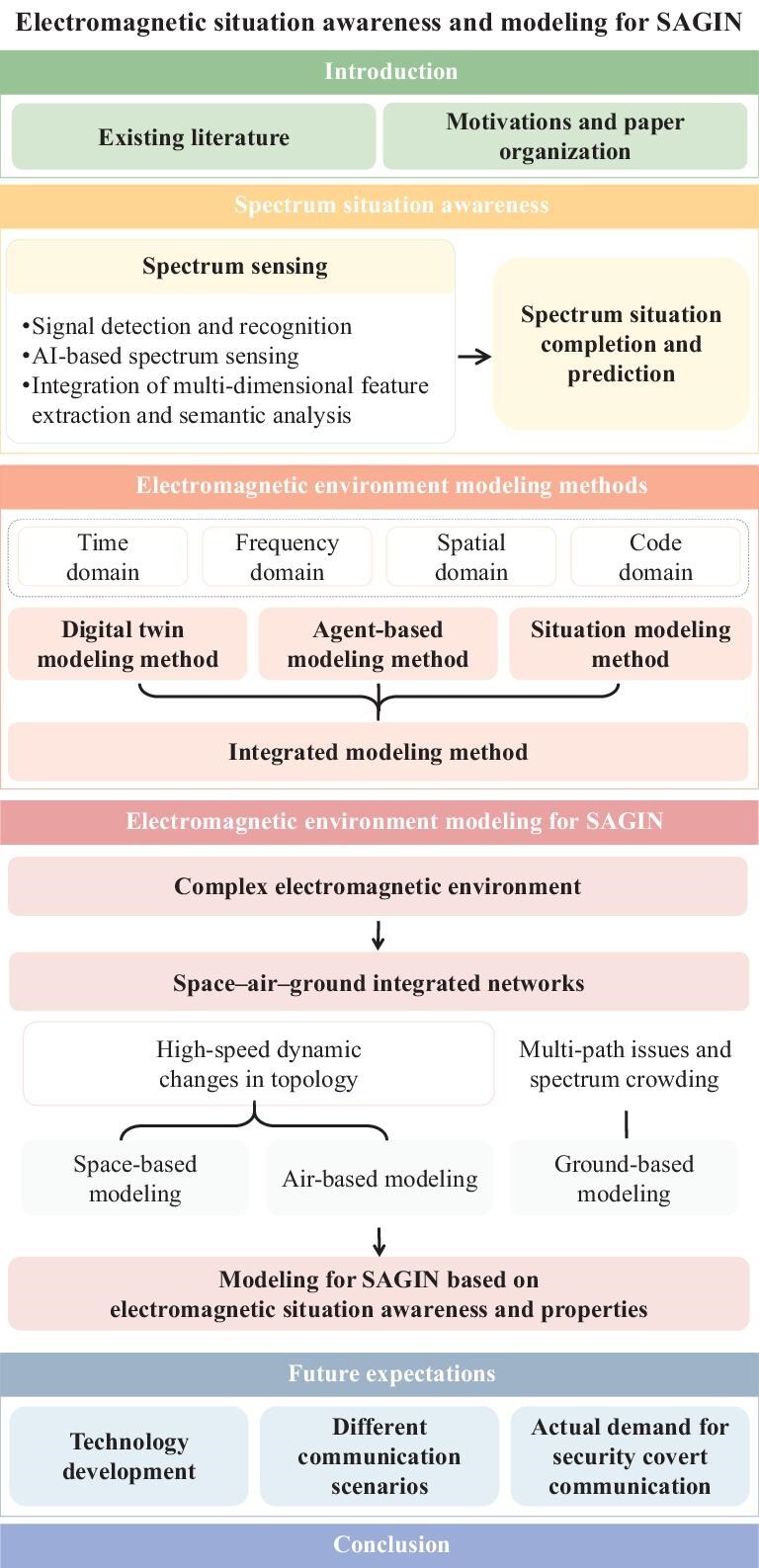
Organization of this paper. In the first section of the paper we provide a comprehensive review of spectrum situation awareness, which summarizes various techniques and recent advancements in spectrum sensing and spectrum situation completion and prediction. In the second section, we present a novel modeling approach across multiple domains, providing a methodological basis for scenario analysis. The third section establishes electromagnetic environment models under various scenarios, taking into account different factors, and facilitates the analysis of the electromagnetic environment in SAGIN networks. In the fourth section, we introduce several relevant concrete communication scenarios to illustrate future expectations for electromagnetic environment modeling. In the final section, we present our concluding remarks on electromagnetic environment modeling.

**Figure 2.**
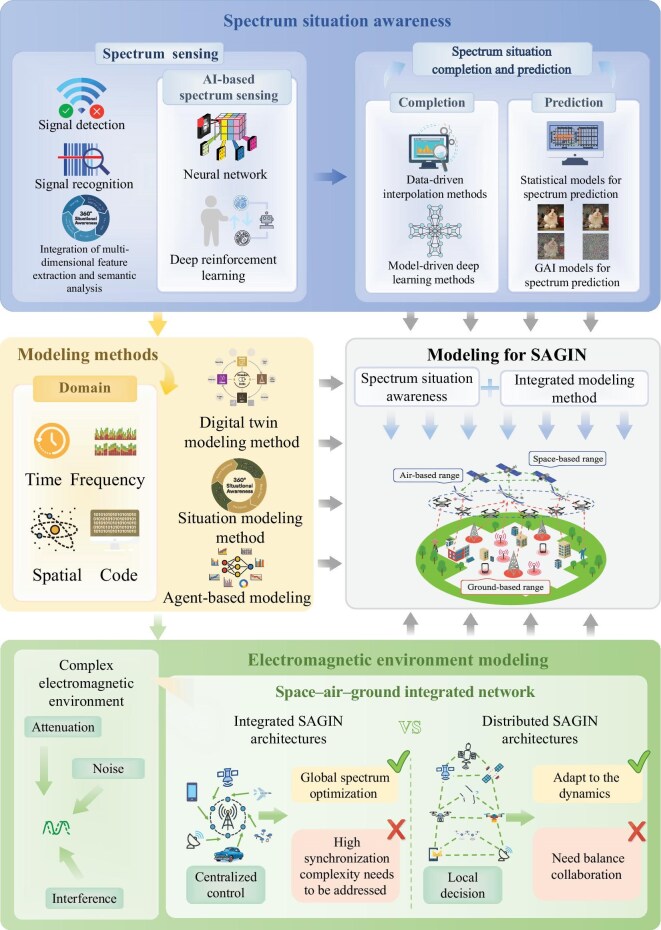
System framework of our work. In SAGIN, spectrum situation awareness provides the foundational data for signal modeling in scene analysis, while modeling methods offer theoretical support. By combining real-time spectrum data with theoretical modeling, scene analysis can more accurately characterize the electromagnetic environment, improving resource allocation, interference management, and overall network performance and reliability.

The urgent need for a comprehensive, up-to-date analysis of the core space–air–ground scenario within SAGIN, excluding maritime and other extensions, has inspired our research (Fig. 1). We aim to enhance the understanding and applicability of electromagnetic environment modeling through the framework in Fig. 2, paving the way for future advancements in the field.

## SPECTRUM SITUATION AWARENESS

**Figure 3.**
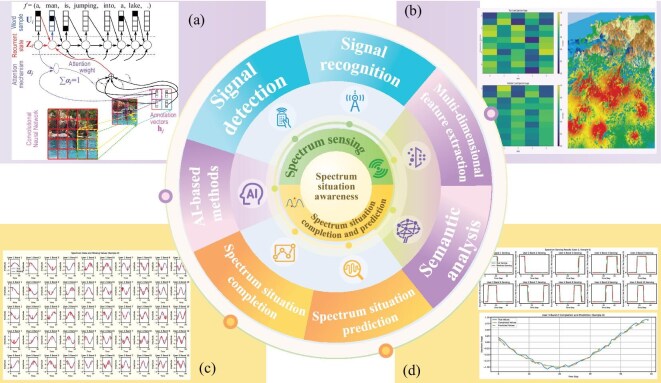
Comprehensive view of spectrum sensing and spectrum situation completion and prediction in spectrum situation awareness. It illustrates this concept with a layered structure: the core represents spectrum situation awareness, followed by spectrum sensing and spectrum situation completion and prediction in the second ring. The outermost layer includes advanced technologies that enhance the capabilities of the inner modules. This structure highlights the interrelated components necessary for effective spectrum management and forecasting. (a) The attention-based encoder-decoder network for describing multimedia content; adapted with permission, copyright: IEEE [31]. (b) The spectrum heat map (left, our experiment) and the electromagnetic situation map (right, adapted from Aiviy Tech [32] with permission). (c) The experimental results of spectrum situation completion (this work). (d) The experimental results of spectrum situation prediction (this work).

Comprehensive spectrum information provides essential data support for electromagnetic environment modeling. In SAGIN, accurate spectrum situation awareness enables the understanding of spectrum distribution and interference conditions in different scenarios [28], laying the foundation for subsequent electromagnetic environment modeling. Therefore, this section introduces spectrum sensing and spectrum situation completion and prediction (Fig. 3), which are essential for obtaining real-time, comprehensive spectrum information and for forecasting future spectrum conditions in complex environments. These techniques are evaluated using the following performance metrics to demonstrate their effectiveness.

- Detection accuracy measures the percentage of correctly identified signals, ensuring effective differentiation between primary user signals and interference.
- Spectrum utilization efficiency is assessed by the reduction of unused spectrum and improved throughput, enabling more efficient resource allocation.
- Interference mitigation evaluates the degree of interference reduction, typically using signal-to-noise ratios (SNRs), with improved awareness aiding dynamic frequency and protocol adjustments.
- Time-to-adapt-to-changes tracks how quickly the system responds to fluctuations in spectrum usage, ensuring fast adaptation to maintain optimal performance and prevent bottlenecks.

By incorporating these metrics into the spectrum sensing, completion and prediction processes, we can quantify the improvements in electromagnetic situational awareness provided by these techniques.

### Spectrum sensing

Spectrum Sensing is key to improving the real-time accuracy of spectrum acquisition and minimizing redundancy in spectrum perception. Its main goal is to capture the current state of the spectrum, including spectrum occupancy, signal modulation, and other relevant information through signal detection and recognition. The integration of multi-dimensional feature extraction and semantic analysis enhances these processes by providing deeper insights into the spectrum environment, allowing for better differentiation between primary user signals and noise. Additionally, AI-based methods play a crucial role in combining traditional detection techniques with advanced data-driven approaches, enabling more intelligent, adaptive, and accurate spectrum sensing.

#### Signal detection and recognition

*Signal detection.* Signal detection is a critical component of spectrum sensing, enabling the identification of signals in the midst of noise and interference [33]. As communication environments become more complex and dynamic, signal detection methods are evolving to meet increasing demands for accuracy, efficiency and adaptability. This part first examines local-node-based signal detection methods and traces the evolution of detection algorithms over time. It then introduces cooperative spectrum sensing (CSS), which leverages the cooperation of multiple nodes to improve detection performance [34].

Local-node-based signal detection methods include the following:

- *Basic signal detection methods.* Energy detection (ED), cyclostationary feature detection (CFD) and matched filter detection (MFD) are key techniques, each with strengths and limitations. ED is simple but sensitive to noise [35,36], CFD is more robust but complex, while MFD offers superior detection at the cost of requiring full signal information. The choice depends on the sensing environment. Signal detection has evolved from basic binary models to advanced approaches addressing noise and interference, with techniques like noise uncertainty adjustments improving detection thresholds and accuracy in fluctuating environments [24].
- *Multiple antenna system.* Multi-antenna systems leverage spatial diversity to improve detection accuracy by mitigating fading and shadowing effects. Techniques such as maximal ratio combining, selection combining and beamforming integrate signals from multiple antennas for better interference suppression, significantly improving robustness in dynamic wireless environments.
- *Two-stage detection process.* This method improves detection accuracy through a sequential process [10]. The first stage uses energy detection for quick assessment, followed by advanced techniques like CFD or fuzzy logic in the second stage to manage uncertainties and enhance reliability.
- *Dynamic threshold.* Dynamic thresholding adapts to changing spectral environments, especially for transient signals. Methods like short-window detection and hidden Markov models [37] (HMM) adjust thresholds based on noise variance, improving performance under varying conditions.

Local-node-based signal detection is cost-effective but struggles in complex environments due to noise fluctuations, multi-path fading and hidden terminals, making it less reliable when signal characteristics are uncertain.

In contrast, CSS enhances detection by allowing multiple sensing nodes to share data, improving signal strength detection, frequency allocation and interference management [34]. It employs two main fusion methods: data fusion and decision fusion.

- *Data fusion.* Data fusion integrates information from multiple nodes to improve spectrum understanding. Various aggregation architectures are explored in cognitive radio networks, such as single cognitive relays [38], multiple relays and multi-hop cognitive sensing, which enhance accuracy, reliability and coverage.
- *Decision fusion.* Decision fusion combines individual detection decisions from nodes to infer the spectrum state. Common rules include ‘*K*-out-of-*N*’, ‘AND’ and ‘OR’, each balancing reliability and simplicity. Meanwhile, fuzzy Logic, a powerful approach in decision fusion, handles uncertainty and imprecision, making it particularly effective in environments with high uncertainty by combining outputs from cognitive radios and SNR data for reliable spectrum sensing outcomes [39].

*Signal recognition.* Signal recognition is crucial in spectrum sensing as it classifies signals to identify their modulation types (e.g. amplitude, frequency, phase) and parameters. Recent research has integrated a new paradigm known as spectrum semantics, which defines the meaningful information and contextual relationships embedded within spectral data, moving beyond simple feature classification to understand the intent and purpose of a signal [40]. By integrating this concept with traditional methods like maximum likelihood estimation and pattern recognition, signal recognition can map signal features to their underlying meaning, thereby enhancing spectrum management and intelligent decision-making.

The maximum likelihood estimation (MLE) method approaches signal modulation recognition as a hypothesis testing problem by comparing likelihoods across modulation hypotheses [41]. Key methods include average likelihood detection, generalized likelihood detection and hybrid detection. Average likelihood detection is efficient and robust but has practical limitations; generalized detection is versatile yet complex and error prone; hybrid detection improves accuracy but challenges real-time performance. While MLE provides high accuracy in ideal conditions, it requires precise models and prior knowledge of modulation types and can be affected by noise and multi-path effects.

Pattern recognition based on feature extraction involves three key steps: preprocessing, feature extraction and classification. Preprocessing improves data quality by reducing noise and inconsistencies. Feature extraction identifies informative features [42], such as statistical measures, energy-based features, cyclic stationary features and wavelet transforms. The final step, classification, uses algorithms like decision trees, Support Vector Machines and neural networks [43].

Pattern recognition offers high computational efficiency and flexibility, but challenges in feature selection and reliance on prior knowledge may limit its effectiveness in diverse scenarios.

#### AI-based spectrum sensing

AI technology has evolved from traditional machine learning (ML) to advanced neural networks and reinforcement learning, transforming spectrum sensing. This progression has emphasized the need for robust data-driven and model-driven methods for practical implementation [44,45]. Neural networks became effective at extracting intricate features, while reinforcement learning enables agents to learn and adapt through interaction with dynamic environments. AI-based methods are now crucial for modern spectrum situation awareness.

*Neural network-based methods.*

- *Convolutional neural networks.* Convolutional neural networks (CNNs) are adapted by their ability to capture spatial dependencies crucial for radio signal processing. They excel at processing multi-dimensional data, effectively identifying complex patterns and distortions that traditional methods overlook, which improves spectrum sensing accuracy in cognitive radio frameworks [46].
- *Recurrent networks.* Recurrent neural networks (RNNs) and long short-term memory networks (LSTMs) are well suited for time-series radio-signal analysis, as they track sequential changes in signal conditions. RNNs maintain a memory of past inputs, while LSTMs address the vanishing gradient problem to retain long-term dependencies [47], which is vital for detecting evolving patterns and anticipating channel availability in dynamic environments [48].
- *Generative models.* Generative adversarial networks (GANs) and variational autoencoders (VAEs) are well suited for addressing data scarcity and anomaly detection in dynamic environments. GANs can simulate radio signals to augment limited datasets, while VAEs learn latent representations to identify rare signal patterns indicative of interference or unauthorized usage [49], making them valuable for unsupervised tasks.
- *Transformer networks*, originally applied in natural language processing, owe their suitability to a self-attention mechanism—one that captures relationships between input elements simultaneously. This enables efficient analysis of large datasets and helps identify critical signal features, making them suitable for real-time spectrum sensing in complex environments [50].

A key limitation of these supervised methods is their reliance on large, high-quality labeled datasets, and their performance may degrade due to concept drift in highly dynamic environments [51].

*Deep-reinforcement-learning-based methods.*

While neural networks have advanced spectrum sensing through pattern learning, they often rely on fixed datasets. In dynamic wireless environments, deep reinforcement learning (DRL) offers a more adaptive solution. In DRL, an agent interacts with its environment, takes actions and receives feedback, optimizing decision-making over time. This adaptability is effective in fluctuating wireless conditions [52], as DRL autonomously determines optimal sensing and access strategies without explicit supervision, outperforming traditional rule-based methods in uncertain environments like cognitive radio networks.

Multi-agent reinforcement learning further enhances DRL by enabling collaboration among agents in distributed sensing systems [49], improving accuracy and efficiency. However, DRL presents significant limitations, including high computational demands, complex state-action spaces and difficulty in designing an effective reward function, which can hinder real-time performance and scalability. Innovations like transfer learning and integration with next-generation networks may offer solutions.

In summary, AI-based techniques, particularly neural networks and reinforcement learning, are transforming spectrum sensing by improving signal detection and management, paving the way for more efficient spectrum situation awareness.

#### Integration of spectrum sensing methods

*Multi-dimensional feature extraction.* Recent advances in signal detection highlight the importance of multi-dimensional feature extraction, which transforms raw spectrum data into actionable spectrum semantics. The goal is to analyze electromagnetic features across various dimensions—such as signal transformation domain statistics (Fourier, wavelet transforms) and signal parameter statistics [53] (frequency, amplitude, phase)—to support tasks like target semantic extraction and radiation source identification. By extracting multi-dimensional features, systems can detect not only the presence of signals but also their contextual significance within the broader spectrum environment.

*Semantic analysis.* Semantic analysis interprets relationships and patterns within detected signals to map electromagnetic features to meaningful spectrum semantics, automating tasks like identifying primary users, interference sources or environmental noise through machine learning or rule-based inference. For example, a high-power, periodic signal feature with a specific modulation type might be semantically identified as a primary user, while a low-power, intermittent signal could be labeled as interference or noise. Integrating semantic analysis with feature fusion enhances detection robustness by combining multi-dimensional feature extraction with contextual understanding, enabling more accurate and adaptive spectrum sensing for tasks such as spectrum occupancy prediction, dynamic spectrum access and interference mitigation [54].

However, despite its advantages, spectrum sensing has limitations. The dynamics and complexity of electromagnetic environments, coupled with sensor limitations, can result in incomplete, inaccurate and discrete spectrum data. Therefore, there is a need for spectrum situation completion and prediction techniques to fill in these gaps and forecast future spectrum conditions, ensuring more accurate and efficient electromagnetic environment modeling.

### Spectrum situation completion and prediction

To address these limitations, spectrum situation completion and prediction techniques play a crucial role. By utilizing advanced algorithms and data-driven models, these technologies aim to bridge the gap in incomplete or uncertain spectrum data and provide a more complete and highly accurate view of spectrum situations.

#### Spectrum completion

Spectrum situation completion fills in missing or incomplete spectrum information by leveraging existing data and predictive models, enabling a comprehensive understanding of spectrum usage, particularly in areas lacking reliable real-time measurements. Techniques for this process include data-driven interpolation and model-driven deep learning methods, both of which use available data to infer and predict missing information based on patterns and trends [54].

*Data-driven interpolation methods.* Interpolation methods estimate missing spectrum data based on available data points, which are commonly used to address spatial and temporal gaps. Techniques such as spatial interpolation [55] and Kriging rely on nearby data to predict missing values, ensuring smooth transitions and minimizing errors.

However, interpolation methods face challenges, particularly with data sparsity and uneven distribution. Large data gaps or non-linear spectrum environments may lead to inaccurate predictions. These methods often assume linear relationships, which may not capture complex spectrum dynamics. Despite these limitations, interpolation remains widely used for simpler spectrum completion tasks.

*Model-driven deep learning methods.* Deep learning methods, utilizing models such as CNNs and RNNs, are particularly effective for spectrum completion tasks, especially when dealing with data that exhibit complex spatiotemporal dependencies. By training deep neural networks, these methods enable the system to learn the intrinsic patterns of spectrum usage, thereby predicting missing data. For example, CNNs are well suited for handling data with spatial dependencies [56], allowing them to extract features from the spatial distribution of the spectrum, while RNNs and LSTMs are more appropriate for capturing temporal dependencies, enabling them to model the evolution of spectrum usage over time.

The main challenge of deep learning methods is their reliance on large, high-quality training data. In sparse or noisy data scenarios, they risk overfitting and poor generalization. Additionally, real-time inference can be limited in complex, dynamic environments.

#### Spectrum prediction

Spectrum prediction anticipates future spectrum conditions based on historical data and current trends, building on the sensing and completion of existing spectrum information. It typically relies on a combination of statistical methods and GAI methods. These approaches simulate and predict spectrum usage patterns by capturing the underlying statistical distribution of the data, thus improving the accuracy and robustness of predictions. The accuracy of the forecast is critical, especially in dynamic environments. Metrics such as the mean squared error or root mean squared error can be used to quantify the accuracy of these predictions.

*Statistical models for spectrum prediction.*

- *Autoregressive integrated moving average (ARIMA).* ARIMA models forecast time-series data, such as spectrum usage patterns, by analyzing past values and using autoregressive, moving average and differencing techniques. They are particularly effective for capturing short-term trends and periodic behaviors in spectrum data.
- *Hidden Markov models.* HMMs are used to model systems with unobservable states, such as transitions between idle and active spectrum bands. By learning from observed data, HMMs predict future spectrum conditions and estimate the likelihood of state changes in dynamic environments.
- *Gaussian processes (GPs).* GPs are non-parametric approaches that provide predictions with uncertainty estimates. By modeling spectrum data as distributions over functions, GPs offer predictions with confidence intervals, making them valuable for sparse or noisy data scenarios.

*GAI models for spectrum prediction.* GAI techniques, including GANs, VAEs, Transformers and diffusion models, offer powerful tools for spectrum prediction by modeling the complex, non-linear dynamics of spectrum usage.

GAI techniques, including large language models and large multimodal models, offer powerful tools for spectrum prediction by analyzing complex, non-linear dynamics [57]. These models excel at identifying intricate spatiotemporal patterns and implicit semantic relationships in large spectrum datasets. Transformers [58] remain foundational, with their self-attention mechanism enabling them to capture long-range temporal dependencies and produce accurate forecasts in large-scale environments. Additionally, diffusion models [59] refine predictions through iterative denoising, improving accuracy in noisy or incomplete data environments and generating realistic spectrum states under uncertainty. By integrating these large-scale models, spectrum prediction can achieve higher accuracy and robustness.

Spectrum situation awareness, together with spectrum situation completion and prediction, complements spectrum situation analysis, ensuring a more complete and forward-looking understanding of the electromagnetic environment, which is crucial for electromagnetic environment modeling for SAGIN.

## ELECTROMAGNETIC ENVIRONMENT MODELING METHODS

Based on spectrum data from spectrum situation awareness, electromagnetic environment modeling simulates how communication entities interact in dynamic environments, analyzing communication quality, resource allocation and system optimization. However, numerical methods and mathematical models [12] are limited across multiple domains (e.g. time, space, frequency, code)[60], and new modeling techniques are required to enhance accuracy and adaptability in CEME. The integration of digital twin, ABM and situation modeling methods into a unified framework can enable a more comprehensive, dynamic analysis of electromagnetic environments.

### Digital twin modeling methods

As electromagnetic environments become more complex, advanced real-time modeling techniques are needed. While digital twin modeling theoretically enables real-time tracking and dynamic analysis of electromagnetic signals through precise time synchronization [19], its actual real-time capability requires rigorous feasibility analysis. Key challenges stem from the temporal constraints in data acquisition, network propagation and computational processing phases. For instance, digital twins for UAV swarms encounter significant accumulated latencies, which impact synchronization and real-time control in high-density electromagnetic environments [61]. To address synchronization tolerance thresholds, emerging solutions combine adaptive time-stamping protocols with predictive compensation algorithms that preemptively adjust phase offsets in electromagnetic field simulations.

Spatially, digital twin modeling creates a three-dimensional (3D) propagation map of electromagnetic waves, considering geography and physical barriers to optimize signal paths and improve anti-interference capabilities. Temporal-spatial co-simulation frameworks further correlate latency thresholds with spatial propagation dynamics, enabling adaptive compensation for delay-sensitive scenarios. By embedding latency-aware validation metrics, the framework guarantees real-time consistency between virtual models and physical electromagnetic states, supporting mission-critical decision-making.

### Agent-based modeling methods

Agent-based modeling is a powerful tool for simulating CEME in SAGIN. Its decentralized structure allows each agent to represent an electromagnetic entity and adapt dynamically to evolving conditions. This makes ABM ideal for modeling interactions across time, frequency, space and code domains.

1. In the time domain, ABM enables agents to respond to rapid changes like signal fluctuations and interference, making it more effective than static models that capture only long-term averages.
2. In the frequency domain, ABM excels in managing dynamic spectrum environments where agents can operate across multiple frequency bands. It supports frequency allocation, spectrum sharing and frequency hopping, making it ideal for scenarios with limited spectrum resources, such as crowded ground-based systems.
3. In the spatial domain, ABM simulates wave propagation, reflection and scattering in 3D environments [62], such as UAV networks and satellite–ground links, capturing the effects of terrain and obstacles—areas where traditional path-loss models fall short.
4. In the code domain, ABM facilitates efficient code management and interference mitigation in systems like code division multiple access, simulating interactions between agents under different communication protocols.

Integrating ABM with decision-making models like the Markov decision process (MDP) and game theory further enhances its adaptability [63], enabling real-time adaptation and improved predictions in uncertain environments.

### Situation modeling methods

Multi-layer situation modeling integrates data from various sources through multi-dimensional feature extraction and fusion, mapping these features into situational semantics. This approach transforms raw data into understandable descriptions by defining situational semantics as the high-level contextual information that interprets the intent, state and relationships of entities within an electromagnetic environment [64]. By creating an electromagnetic behavior knowledge graph that links these semantics to specific behaviors, this approach builds a dynamic knowledge base that enhances situational understanding and decision-making [65].

Features are extracted across multiple domains using various analytical methods and then fused into a cohesive model. Following this, event detection and semantic mapping transform these features into situational semantics, capturing events, state changes and other key information. Finally, an electromagnetic behavior knowledge graph is built, recording time, frequency, location and code information across domains to create a comprehensive network. While this method ensures accurate perception, prediction and decision support, it is computationally intensive, requiring significant data fusion efforts and facing challenges in real-time processing and non-linear dynamic behavior prediction.

### Integrated modeling methods

Each modeling method possesses distinct advantages and disadvantages. Digital twin modeling excels in accurately reproducing the details of both physical and spectral spaces, thus offering a robust data foundation; however, the scope of related research remains relatively limited. ABM emphasizes decentralized structures that can effectively simulate the complex behaviors and interactions of multiple agents, yet it is characterized by high computational complexity and a strong dependence on initial conditions. Situation cognitive modeling facilitates comprehensive situational understanding and supports real-time decision-making, but it is contingent upon the availability of high-quality real-time data and precise feature extraction.

**Figure 4.**
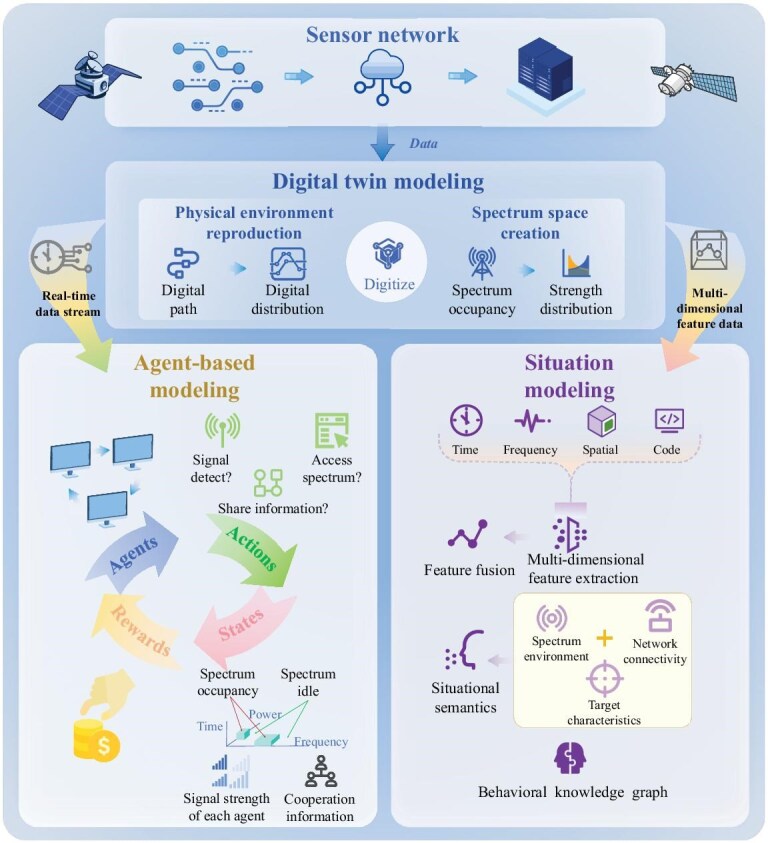
Integrated modeling for CEME. Data collection and processing: a network of multiple sensors collects and processes raw data to provide a foundation for modeling. Virtual environment construction: processed data are used to simulate the electromagnetic spectrum environment in the information space, creating a virtual representation of physical and spectral spaces. Dynamic agent interaction: in ABM, agents interact dynamically according to dataset rules, simulating electromagnetic behaviors. Situational cognition and decision making: situational cognitive modeling extracts features and constructs a knowledge graph for real-time understanding and resource allocation.

Figure 4, combining the digital twin modeling, ABM and situation cognitive modeling, not only enhances the accuracy and adaptability of the model but also provides robust support and optimization strategies for intricate application scenarios, such as spectrum management, radar systems and radio navigation, thereby ensuring efficient and precise decision-making in the ever-evolving electromagnetic landscape.

In CEME-aware SAGIN systems, key challenges include the co-existence of spectrum monitoring and communication networks, workflow optimization and managing overhead from new components [66]. While integrating digital twin, ABM and cognitive situation modeling enhances adaptability, it also introduces system-level complexity requiring careful optimization, particularly concerning computational overhead, real-time feasibility and scalability. Specifically, digital twins demand high-fidelity modeling and frequent state synchronization, leading to substantial computational and communication burdens; agent-based modeling may encounter a combinatorial explosion of interactions as the system scales and cognitive situation modeling imposes high requirements on real-time data processing and analytics. These factors collectively constrain the practical deployment of such integrated systems in large-scale, dynamic environments.

Coordinated operation across these models improves electromagnetic environment representation and streamlines workflow. Digital twins offer real-time feedback, ABM simulates agent behaviors and situation models support dynamic adjustments to protocols and resource allocation. This synergy enables efficient coexistence of spectrum monitoring and communication tasks, reduces latency and optimizes throughput via adaptive resource sharing.

However, these benefits come with computational and communication overheads. Digital twins require frequent synchronization and real-time signal analysis, while ABM increases complexity through decentralized multi-agent coordination across various domains [67]. Quantitative analysis of these overheads is essential to balance system performance and resource consumption.

The integrated modeling approach, while improving situational awareness and system agility, still requires addressing workflow and overhead challenges to achieve efficient, scalable SAGIN deployment in dynamic electromagnetic environments.

## ELECTROMAGNETIC ENVIRONMENT MODELING FOR SPACE–AIR–GROUND INTEGRATED NETWORKS

**Figure 5.**
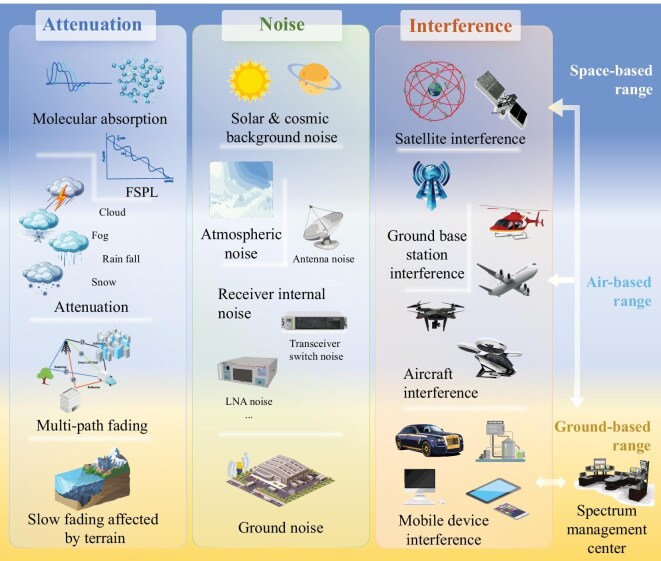
The influencing factors of CEME, including space-, air- and ground-based ranges, vary across different scenarios. The dynamic topologies of satellites, aircraft and drones, with constant changes in position, velocity and altitude, challenge network stability and performance. To address these issues, targeted space-based and air-based models are needed to adapt to these evolving conditions. In comparison to space and air, ground communication has become increasingly prevalent due to the proliferation of terminals, resulting in significant multi-path issues. Analyzing attenuation, noise and interference is crucial for understanding the electromagnetic characteristics in these scenarios.

In the previous section, we provided a detailed introduction to the methods and theoretical foundations of electromagnetic space modeling. Building on this foundation, this section will delve into the similarities and differences of electromagnetic spectrum environments across various scenarios, as shown in Fig. 5, as well as how these characteristics impact our modeling strategies and resource management decisions.

### Complex electromagnetic environment

The electromagnetic environment has become increasingly complex due to the rapid proliferation of diverse electromagnetic technologies, leading to what is now known as CEME [68]. Characterized by intangibility, diversity, antagonism, dynamism and relativity, CEME arises from the interactions of space, frequency, time and energy across four dimensions: spatial distribution, frequency spectrum, time variations and electromagnetic intensity.

The causes and characteristics of CEME are reflected in a multi-level, multi-dimensional and dynamic system.

- *Diversity of communication systems.* Communication systems range from terrestrial to space-based applications [69], including geosynchronous Earth orbit (GEO), medium Earth orbit, low Earth orbit (LEO) satellites, and specialized systems, providing global coverage for communication and navigation. However, frequency overlap and interference are common challenges. Civil aviation, UAVs, high-altitude platforms and low-altitude systems like flying cars also share frequency bands, leading to conflicts [70]. Additionally, terrestrial systems face interference due to differing frequencies and standards.
- *Complexity of electromagnetic propagation.* Advancements in technology and urbanization have increased the complexity of electromagnetic propagation [71]. Signals now traverse environments filled with obstacles, encountering reflections and interferences from buildings, devices and atmospheric changes, making propagation increasingly unpredictable.
- *Diversified frequency band usage.* The frequency spectrum has expanded significantly, encompassing a wide range of frequencies used across various applications [72], including radio broadcasting, mobile communication, satellite communication, radar and wireless local-area networks. As the demand for frequency spectrum grows, the allocation and use of frequency bands have become more flexible, requiring careful coordination to manage conflicts and ensure electromagnetic compatibility.
- *Expansion of electromagnetic signal propagation space.* Electromagnetic signal propagation now spans from near-Earth to outer space, covering LEO to GEO satellites. This expands the complexity of the electromagnetic environment, as signals interact with diverse terrains, weather conditions and longer distances. Propagation is affected by multi-path effects, satellite motion and varying orbital environments, creating a multi-level, multi-dimensional environment with unique characteristics and interference factors at each layer [73].
- *Diversification of the electromagnetic signal function.* Technological advancements have expanded the functionality of electromagnetic signals beyond traditional communication to include high-definition video, intelligent control and remote sensing [74]. Signals now support basic communication, internet access and high-precision navigation services like time synchronization and speed measurement. This functional diversification enhances the utility of signals while simultaneously increasing the complexity of the electromagnetic environment.

Understanding the causes and impacts of CEME is crucial for improving signal quality and supporting SAGIN development. To address CEME’s dynamic challenges, a holistic and adaptive spectrum monitoring architecture is essential. This framework integrates various technologies to enhance detection accuracy, spectrum utilization and interference mitigation [75].

- *Sensor networks.* Ground, airborne and spaceborne sensors (e.g. software-defined radios, spectrum analyzers) form a distributed network that monitors multi-band electromagnetic activity, ensuring broad coverage.
- *Satellite monitoring.* Satellites conduct real-time spectrum scanning, bridging gaps between ground and aerial layers.
- *UAVs and high-altitude platforms.* UAVs and high-altitude platforms (HAPs) enhance coverage in interference-prone regions, especially urban areas, acting as mobile or persistent sensing nodes within the sensor network.
- *Centralized fusion platform.* Data collected by the distributed sensor network (including ground stations, UAVs/HAPs and satellites) is aggregated in real-time by a central platform for coordinated spectrum management.

Real-time data fusion is critical, integrating inputs from sensors (e.g. signal strength, interference, weather) using frameworks like Apache Kafka and Spark [76]. Advanced filters (e.g. Kalman, particle) remove noise and produce accurate electromagnetic models. Based on this, machine learning supports real-time decisions on spectrum scheduling and interference mitigation. Intelligent systems further predict CEME dynamics using historical and live data, enabling proactive, stable network operation.

### Space–air–ground integrated networks

In developing effective space–air–ground integrated networks, it is important to recognize that the complex causes and characteristics discussed contribute to signal attenuation, noise and interference. To address these issues, it is necessary to construct and refine different models tailored to specific emphases.

Signal attenuation in electromagnetic wave propagation is caused by reflection, scattering and refraction, and is categorized into large-scale (path loss) and small-scale attenuation (multi-path effects) [77]. Noise, both internal (thermal) and external (natural and man-made), degrades signal quality and reliability [78]. Electromagnetic interference [79] from systems like communication or radar can be malicious (e.g. electronic warfare) or non-malicious (e.g. equipment faults) and is classified into space-based, air-based and ground-based types.

To effectively address signal attenuation and interference in space, air and ground environments, tailored models must be developed for each scenario. These models enable precise evaluation of signal loss, noise and interference based on specific propagation conditions. A layered and integrated analysis will provide robust theoretical and technical support for the efficient operation of space–air–ground integrated networks.

The distinct propagation physics, interference profiles and mobility dynamics across space, aerial and terrestrial domains mandate domain-specific models. Space faces extreme path loss and cosmic noise; aerial platforms contend with atmospheric effects, Doppler shifts and weather fading; terrestrial networks grapple with dense multi-path, shadowing and heterogeneous interference. To accurately capture these domain-specific electromagnetic signatures—including their inherently different topological dynamics driven by unique environmental and mobility constraints—we establish integrated models for three core scenarios: space-based modeling, air-based modeling and ground-based modeling, as shown in Table 2.

**Table 2.** Comparison of key electromagnetic characteristics.

Parameter	Space-based	Air-based	Ground-based
Attenuation	• Free-space loss	• Free-space loss	• Strong multi-path
	• Atmospheric absorption	• Rain attenuation	• Shadowing
	• Rain (Ka+ bands)	• Multi-path reflections	• Penetration loss
Noise sources	• Cosmic background	• Environmental noise	• Environmental noise
	• Solar radiation	• Atmospheric	
Interference	• Satellite cross-links	• Aircraft congestion	• Co-channel devices
	• Ground station misalignment	• Satellite signals	• Malicious jamming
Mobility	• High orbital dynamics	• Rapid 3D movement	• User mobility
		• Doppler effects	• Static infrastructure
Propagation	• Near-vacuum environment	• Atmospheric turbulence	• NLOS dominant
	• Long distances	• Medium distances	• Urban clutter
Key challenges	• Signal delay	• Channel stability	• Interference coord.
	• Precision pointing	• Weather sensitivity	• Coverage holes
Modeling complexity	High	High	Medium high

#### Space-based modeling

The space-based modeling approach is tailored to a satellite-specific perspective, allowing for the accommodation of rapid changes in satellite positions and their relative relationships.

- *Attenuation and noise.* Satellite communication systems involve direct links, but satellite-to-ground and inter-satellite links have distinct attenuation and interference characteristics. Satellite-to-ground links experience free-space loss, multi-path effects, molecular absorption and weather-related attenuation, categorized into large-scale (distance-dependent) and small-scale effects (modeled by distributions like Nakagami [80]). Noise sources include internal receiver noise, cosmic background noise, solar noise, ground noise and atmospheric noise. Inter-satellite links primarily face free-space attenuation and similar noise sources, including cosmic background and solar noise.
- *Interference.* Space interference in satellite communication systems includes uplink and downlink interference, both affected by the dynamic satellite topology [81]. Uplink interference occurs when Earth stations accidentally transmit to nearby satellites due to their rapid motion. Downlink interference happens when a satellite’s transmission affects adjacent Earth stations within the same frequency band, caused by insufficiently narrow antenna beams unable to reject signals from nearby satellites. Airborne interference is influenced by the dynamic satellite topology [82]. Uplink interference arises from electromagnetic radiation emitted by ground equipment or aerial platforms, especially when the source and uplink frequencies are close, and satellite movements increase interference. Downlink interference, caused by radio equipment or metallic structures on aerial platforms, can affect a larger area due to reflections and scattering from high-altitude antennas, worsened by satellite motion. Ground interference arises from frequency overlap, high signal strength and network congestion [83]. When ground radio transmitters operate close to satellite frequencies, strong ground signals can overwhelm weaker satellite signals, reducing communication quality. In high-density areas, network congestion competes for bandwidth, lowering satellite communication efficiency. Frequent satellite movement further complicates the management of ground interference.

#### Air-based modeling

The rapid movement of airborne platforms, like airplanes or drones, causes fluctuating communication links with the ground, influenced by attenuation and interference, resulting in constant signal quality variations.

- *Attenuation and noise.* Aerial platforms, like aircraft and drones, face significant challenges due to weather and signal attenuation, including free-space loss, multi-path effects, absorption by atmospheric molecules, diffraction and scattering [84]. Unlike deep-space communication, air-based systems also contend with internal receiver noise and external environmental interference. Attenuation and noise significantly impact both uplinks and downlinks for aerial platforms. Atmospheric effects, including molecular absorption and especially rainfall attenuation, are major contributors to signal degradation in both directions [85]. Additionally, for downlinks, ground thermal noise becomes a significant factor affecting reception quality at the aerial receiver [86]. The dynamic movement of aerial platforms further complicates signal propagation, altering attenuation and noise levels as positions and altitudes change.
- *Interference.* Air-based interference impacts communication links, especially with the increasing number of high-altitude aircraft and drones. Electromagnetic interference (EMI) occurs when frequencies overlap in congested airspace. Environmental obstacles and weather can cause signal reflection, multi-path interference and instability. Intentional jamming or electronic warfare tactics may also disrupt communication, compromising system integrity. Satellite interference challenges air-based communication links, especially as the number of satellites increases. Overlapping frequencies can disrupt aircraft communication, with signals reflecting or scattering in the atmosphere, degrading reception. Additionally, interference between multiple satellite systems further impacts aircraft communications. Ground interference affects ground-to-air communication links, particularly with the rise of high-power ground radars [87]. This disrupts airborne systems, while congestion in high-density areas like urban environments or near airports further competes for bandwidth, reducing communication efficiency.

#### Ground-based modeling

Ground-based communication channels are highly variable due to environmental conditions, topography and obstacles like buildings. These factors cause significant fluctuations in signal attenuation, noise and interference, increasing challenges and uncertainties in signal transmission.

- *Attenuation.* Electromagnetic wave propagation for communication involves direct, sky and ground waves. While these propagation modes are most prominent in ground-based networks due to terrain and ionospheric interactions, similar mechanisms also manifest in space-air communications, albeit with distinct characteristics. Direct waves travel in a straight line, experiencing free-space loss. Sky waves are reflected by the ionosphere and face ionospheric absorption and weather-related attenuation, varying by time of day. Ground waves travel along the Earth’s surface, affected by conductivity and topography, leading to absorption and diffraction losses. Each wave encounters distinct forms of attenuation, shaped by its unique propagation characteristics and the environmental factors it interacts with [88].
- *Noise.* Ground-based communication, both uplink and downlink, is impacted by natural and man-made noise. Man-made noise from activities like machinery, traffic and industry interferes with both the environment and communication systems, reducing signal clarity and communication quality.
- *Interference.* Ground-based interference affects communication systems through EMI and radio-frequency interference from sources like wireless base stations, radar, power lines and electronic devices. This leads to signal degradation, interruptions, delays and a reduced user experience. The interference of space-based communication systems with ground communication networks includes signal overlap, EMI disrupting ground equipment and dynamic interference from satellite mobility. Additionally, antenna sidelobe effects can allow interference, reducing reception quality. This phenomenon is universal across space-, air- and ground-based systems but is particularly acute in dense ground networks due to proximity and multi-path effects. Similarly, airborne communication systems, like aircraft and drones, interfere with ground systems through spectrum interference and mechanical noise [75]. Aircraft radar and communication equipment can disrupt ground receivers, while engine noise obscures weak signals. Airflow and turbulence from aircraft also reduce the precision of ground weather radar.

### Modeling for SAGIN based on electromagnetic SA and properties

#### Comparative analysis of integrated versus distributed SAGIN architectures

By contrasting integrated and distributed SAGIN architectures, we can better understand how CEME dynamics influence trade-offs in complexity, signaling and efficiency. These trade-offs are critical for balancing global optimization and local adaptability in resilient, context-aware system design.

- *Network complexity.* Integrated architectures optimize resources via centralized control but face high coordination complexity due to real-time synchronization across heterogeneous nodes. Distributed architectures reduce this by enabling local autonomy (e.g. UAV-based routing), but require mechanisms to balance independence and cross-domain collaboration under CEME variations.
- *Signaling overhead.* Integrated systems incur significant signaling due to frequent control-node interactions, which scale poorly. Distributed approaches reduce global signaling through local decisions but risk hidden overhead from resolving redundant conflicts. The key trade-off is between centralized scalability and distributed overhead ambiguity.
- *Operational efficiency.* Integrated architectures offer high global efficiency in stable environments but lack responsiveness. Distributed systems adapt quickly to local CEME changes (e.g. UAV swarm beamforming) but may suffer from fragmented resource allocation. Bridging this gap requires lightweight coordination to balance global and local performance.

#### Holistic modeling for SAGIN

**Figure 6.**
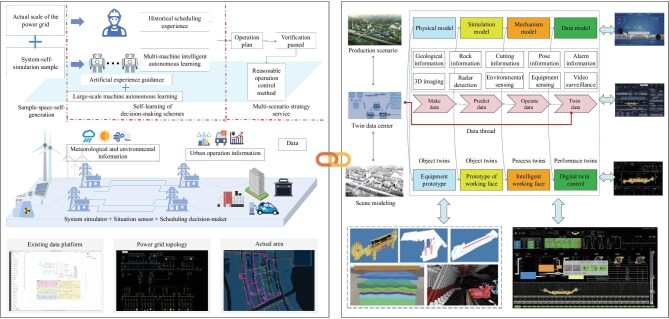
Industrial case studies: in preliminary industrial validations at power plants (left, reproduced from [89] with permission, copyright: State Grid Corporation of China) and mining operations (right, reproduced from [90] with permission, copyright: China Coal Society), the three modeling methods were integrated to analyze complex electromagnetic environments. Digital twin modeling created high-fidelity virtual replicas of the physical infrastructure, including antenna layouts and signal paths. Agent-based modeling simulated the dynamic behavior of individual devices and operators, capturing how they adapt to and cause interference. Situation modeling then fused this real-time data to provide a holistic, multi-layered view of the electromagnetic environment, enabling precise interference analysis and spectrum optimization under dynamic conditions.

To address the complex multi-path interactions across space, air and ground domains—such as satellite-to-UAV interference and terrestrial-to-satellite uplink interference—we propose a dedicated cross-domain interaction modeling framework. This framework integrates three core components: digital-twin-based propagation coupling for high-fidelity signal path emulation, ABM for dynamic interference adaptation and multi-layer situation modeling for holistic electromagnetic awareness. Crucially, this approach incorporates CEME awareness to evaluate signal attenuation, noise and cross-network interference, while systematically managing trade-offs between integrated and distributed architectures [91]. Notably, classic cases in Fig. 6 have demonstrated the feasibility of integrated electromagnetic modeling.

**Figure 7.**
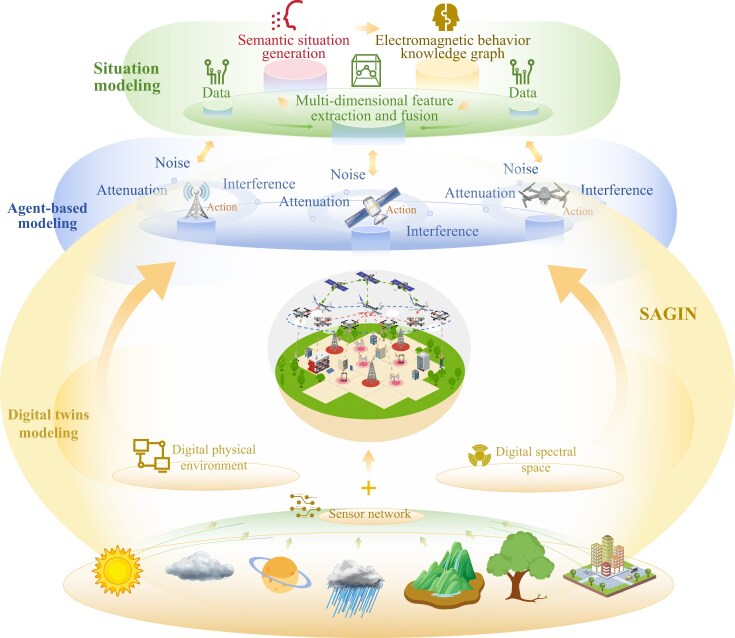
Three-in-one modeling strategy for SAGIN: digital twin modeling replicates physical and spectral spaces, simulating environmental influences like weather and terrain on electromagnetic signals, wave propagation and interference. This simulation feeds data into agent-based modeling, where tools such as game theory and Markov decision processes optimize the actions of agents in competitive and cooperative scenarios, managing resources like spectrum and power. The interaction between agents, including noise and interference, is analyzed through concepts like the Nash equilibrium for resource management. Situation modeling further enhances the process by integrating real-time data from digital twins and network nodes, synthesizing complex information into a comprehensive understanding of the network’s state. This creates a dynamic electromagnetic behavior knowledge graph that supports macro-level spectrum regulation and continuously improves agent decision-making through feedback.

The integration of digital twin modeling, ABM and multi-layer situation modeling (Fig. 7) addresses SAGIN’s multi-level challenges. Digital twins emulate satellites, drones and ground stations for centralized interference control. ABM enables autonomous, adaptive node-level behavior in distributed architectures. Multi-layer modeling balances centralized efficiency and decentralized resilience, with CEME awareness guiding real-time trade-offs.

Simulation data from digital twins and real-world spectrum awareness support behavior optimization using tools like game theory and MDP. In ABM, attenuation, noise and interference are modeled as competitive games involving agent perception, decision-making and adaptability [92]. Game theory helps analyze strategies under resource constraints, such as spectrum and power limitations, with the Nash equilibrium ensuring stable agent behaviors in dynamic environments.

The situation modeling system fuses high-precision digital twin data with multi-level node information. Through multi-dimensional data extraction and fusion, it constructs a semantic understanding of the network and maps it across physical and spectral domains. This forms a comprehensive electromagnetic behavior knowledge graph, supporting macro-level spectrum regulation and enabling agents to optimize strategies through feedback. In a recent smart-grid deployment case, the system was evaluated across four key dimensions: interpretability, predictability, interactivity and scalability—setting benchmarks for SAGIN applications.

This three-in-one modeling strategy significantly improves network intelligence, resource efficiency and spectrum management, making SAGIN more adaptive, secure and efficient in complex conditions.

## FUTURE EXPECTATIONS

The development of spectrum situation analysis and electromagnetic environment modeling underscores their potential for advancing technology, modeling methods and communication scenarios. As wireless technologies and network complexities grow, integrated approaches to spectrum sensing and modeling are essential. Advances in RF communications and AI support optimized spectrum usage but must be adapted to specific scenarios, such as space–air–ground–sea networks and the Internet of Vehicles (IoV), each presenting unique challenges. Additionally, the demand for secure communications drives the need for advanced models to ensure privacy and secure transmissions. These factors collectively shape the future of modern communication systems.

### Technology development

In the rapidly evolving wireless communications landscape, spectrum situation analysis and electromagnetic environment modeling face new challenges and opportunities. To improve spectrum efficiency and adapt to dynamic conditions, integrated technological development is essential.

#### High-frequency band communication technology

High-frequency band communication, particularly in the terahertz range, offers promising benefits such as higher bandwidth, faster transmission and greater spectrum capacity, with potential applications in smaller devices and reduced latency. However, challenges like weak signal penetration and significant path loss [93] highlight the need for dynamic spectrum sensing and effective electromagnetic modeling to minimize interference and enhance communication efficiency. Additionally, advancements in hardware, network infrastructure and physical layer technologies are essential for the successful implementation of terahertz communication systems.

#### Artificial intelligence technology

AI is revolutionizing wireless communication, with future advancements poised to enhance system performance significantly [94]. As AI technologies evolve, they will enable more intelligent spectrum management, real-time environmental sensing and adaptive interference mitigation. ML algorithms will increasingly optimize network traffic, predict spectrum demand and enable seamless resource allocation. GAI, such as GANs, will further refine interference management and power control strategies [95]. Looking ahead, AI will enable communication systems to autonomously adjust to changing conditions, improving efficiency, reducing latency and enhancing reliability, ultimately driving the next generation of intelligent, self-optimizing wireless networks.

### Advancements in modeling methodologies

Future electromagnetic environment modeling requires breakthroughs in methodological innovation to address SAGIN’s complexity.

#### Hybrid multi-scale modeling

Integrating quantum-scale device noise with network-level interference through cross-layer physics-informed neural operators enables real-time simulation of satellite-terrestrial signal coupling. This approach bridges atomic-level quantum effects and network-level dynamics, crucial for predicting interference in heterogeneous SAGIN architectures.

#### GAI for synthetic data

Leveraging diffusion models enables the generation of high-fidelity electromagnetic scenarios for training robust AI models under data-scarce orbital conditions. These synthetic datasets can overcome the limitations of real-world data collection in space environments, enhancing model generalization.

#### Semantic-aware cognition

Intent-driven modeling combines knowledge graphs with reinforcement learning to dynamically reconfigure networks based on inferred user objectives. This enables autonomous adaptation to mission-critical requirements without human intervention.

### Scenario-driven modeling requirements

Different scenarios require tailored spectrum situation analysis and electromagnetic environment modeling. Integrating spectrum sensing with modeling and optimizing these approaches for specific applications is key to efficient spectrum use and reliable communication.

#### Cross-domain heterogeneous dynamic integration networks

The future development of cross-domain heterogeneous dynamic integration networks lies in achieving seamless connectivity and coordination across air, space, ground and maritime domains [96], creating a unified and intelligent communication ecosystem. These networks must support multiple communication protocols and platforms, enabling real-time data exchange and resource optimization in highly dynamic environments. For example, in military applications, such a network could integrate ground-based communication systems with UAVs, satellite communication systems and maritime assets, allowing for real-time coordination during missions involving complex geographical and operational conditions. This integration must address challenges stemming from system heterogeneity, dynamic spatiotemporal variations and complex electromagnetic environments. Spectrum sensing and electromagnetic environment modeling are critical for addressing these issues. Accurate sensing optimizes resource use and minimizes interference, while precise modeling supports network design and adaptation to diverse environments. In scenarios like disaster recovery, this technology could ensure efficient communication across diverse platforms, enhancing response times and coordination between teams on the ground, in the air and at sea.

#### Multi-domain IoV networks

Multi-domain IoV networks, which integrate ground vehicles, low-altitude aircraft and satellites, form a dynamic mobile network where real-time communication and spectrum management are crucial [97]. These networks enable vehicles, drones and aerial platforms to communicate seamlessly, supporting applications like autonomous driving, traffic management and delivery services. For example, autonomous vehicles could coordinate with drones and satellites to share data on road conditions and hazards, improving safety and traffic flow. Spectrum situation analysis and dynamic management enable interference avoidance and efficient spectrum utilization, addressing challenges like non-stationary channels [98] and communication congestion. Future advancements will focus on AI-driven algorithms for real-time monitoring, resource optimization and interference detection. For instance, in urban environments, AI could dynamically allocate spectrum resources to prioritize communication between autonomous vehicles and traffic control centers, while minimizing interference with surrounding communication systems. This will enhance adaptability, security and seamless data exchange across the ground and aerial networks, supporting the advancement of intelligent vehicle systems.

### Actual security covert communication

Covert communication is crucial for information security and privacy [99], especially amid rising data volumes and interference threats. Spectrum situation analysis enables real-time security risk detection while minimizing exposure, demanding intelligent algorithms and robust mechanisms to simultaneously ensure concealment and protection. ABM is vital for monitoring the electromagnetic environment, enabling quick threat detection and response adjustments. Future advancements should leverage multi-agent game theory [100] to enhance cooperative/competitive agent interactions, thereby improving threat detection and system security.

However, achieving verifiable covertness in SAGIN faces a fundamental trilemma among covertness, reliability and rate.

- *Covertness-reliability trade-off.* Reducing transmit power lowers detection probability but degrades SNR, increasing bit errors. Conversely, power boosting improves reliability at the cost of detectability, governed by fundamental limits like the square-root law.
- *Covertness-rate conflict.* Techniques like spread spectrum or artificial noise enhance LPI but sacrifice spectral efficiency. Adaptive waveform design must balance stealth and throughput under dynamic SAGIN channel fading.
- *SAGIN-specific complexity.* Multi-domain operations intensify trade-offs. Satellite–ground links suffer predictable propagation delays, easing detection, while UAV mobility introduces Doppler shifts that may mask signals but disrupt synchronization.

This requires balancing antagonistic constraints: power reduction for stealth degrades SNR and spectral efficiency, while reliability demands conflict with covertness guarantees under dynamic cross-domain channel conditions.

## CONCLUSION

In the 6G era, the demand for higher data rates, lower latency and ubiquitous connectivity is growing, but the radio spectrum is limited. This makes accurate spectrum situation analysis and electromagnetic environment modeling crucial for efficient spectrum usage.

This paper explores recent advancements in spectrum situation awareness, emphasizing the importance of spectrum sensing and prediction. Then, we examine how integrated environment modeling approaches, such as digital twin, ABM and cognitive modeling, enhance accuracy and adaptability in CEME. Additionally, we craft tailored electromagnetic environment models for diverse scenarios in SAGIN, considering factors like attenuation and interference. Finally, our exploration identifies promising future research directions, including technology development, specific communication scenarios and actual demand for security covert communication.
